# P01-020 – Starting time of inflammatory attacks in patients

**DOI:** 10.1186/1546-0096-11-S1-A24

**Published:** 2013-11-08

**Authors:** F Berktaş, N Alpay, B Toz, OK Bakkaloğlu, B Erer, A Gül

**Affiliations:** 1 Medicine and Life Sciences, Faculty of Health, Maastricht University, Maastricht, the Netherlands; 2 Department of Internal Medicine, Division of Rheumatology, İstanbul University, İstanbul, Turkey; 3 Department of Internal Medicine, İstanbul Medical Faculty, İstanbul University, İstanbul, Turkey

## Introduction

Familial Mediterranean fever (FMF), the most common of the hereditary autoinflammatory diseases, is characterized by recurrent self-limiting attacks of fever and/or serositis accompanied with acute phase response. Recurrence of attacks does not show a clear periodicity, and its timing is usually unpredictable. Little is known about the factors triggering or precipitating the attacks, and some patients describe physical or emotional exertion, menstrual cycle and dietary changes as possible triggers of the attacks. In other hereditary autoinflammatory disorders, a diurnal variation for the attack was observed with a tendency to experience attacks during evening or night. Recent data suggest that expression of some genes may show a circadian rhythm and affect the immune system, especially innate immune response.

## Objectives

In this study, we aim to collect data retrospectively from FMF patients about the starting time of their attacks.

## Methods

As a pilot study, we did a questionnaire based survey in 113 consecutive adult FMF patients. All patients fulfilled the Tel-Hashomer criteria for the diagnosis of FMF, and experienced attack(s) during the last year. All patients were interviewed directly or by telephone contacts to answer the questionnaire items about their attacks. The list of questions included usual start time of attacks during the day, their attack frequency, severity and possible triggers (such as sleeplessness, hunger, tiredness, stress, diet, medications, other diseases, menstruation and cold exposure) of the attacks during the past year.

## Results

All patients (n=113) agreed to participate in the study and provided answers to the questions. Their mean age was 34.6 and sixty-two (55%) were female. The most commonly reported attack triggering factors were emotional stress (59%), menstruation (53% of female patients), tiredness (49%), followed by dietary changes (22%), cold exposure (15%), sleeplessness (10%) and hunger (4%). Only 9 patients (8%) had an occupation with night shifts. In all group, 76% of the patients provided a definite answer to the question about starting time of attacks. The majority reported that of their attacks started in the evening (41%), and less frequently in the morning (19%), at night (11%) and in the afternoon (4%). There was no correlation between the start time of the attacks and triggering factors or night shifts.

## Conclusion

This questionnaire-based retrospective survey suggests that starting time of FMF attacks have a tendency for evening. This information may provide some clues about circadian changes affecting inflammation and attack tendency, after confirmation with prospective data collection.

## Disclosure of interest

None declared.

